# Estimation of Shipping Emissions in Developing Country: A Case Study of Mohammad Bin Qasim Port, Pakistan

**DOI:** 10.3390/ijerph191911868

**Published:** 2022-09-20

**Authors:** Iftikhar Hussain, Haiyan Wang, Muhammad Safdar, Quoc Bang Ho, Tina D. Wemegah, Saima Noor

**Affiliations:** 1 School of Transportation & Logistics Engineering, Wuhan University of Technology, Wuhan 430063, China; 2 Intelligent Transportation Systems Research Center, Wuhan University of Technology, Wuhan 430063, China; 3 National Engineering Research Center for Water Transport Safety, Engineering Research Center for Transportation Safety, Wuhan 430063, China; 4 Department of Science and Technology, Vietnam National University-Ho Chi Minh City (VNU-HCM), Ho Chi Minh City 700000, Vietnam; 5 Department of Civil Engineering, Accra Technical University, Accra P.O. Box 561, Ghana; 6 Karakoram International University, Gilgit 15100, Pakistan

**Keywords:** global warming, climate change, transportation emissions, shipping emissions, air pollution, Sustainable Development Goals, Pakistan port

## Abstract

Transportation has the highest dependence on fossil fuels of any sector and accounts for 37% of carbon dioxide (CO_2_) emissions. Maritime transportation is responsible for around 940 million tons of CO_2_ and approximately 3% of global emissions annually. The significant increase in shipping activities around the globe has magnified the generation of toxic pollutants. In recent years, shipping emissions have received significant attention in developed countries due to global climate change, while in developing countries, researchers are making enormous efforts to tackle this catastrophic and pressing issue. This study considers Muhammad Bin Qasim Port (MBQP), Karachi, Pakistan as a case study. This study employed an activity-based or bottom-up approach with a standard procedure to estimate the various anthropogenic pollutants emissions including particular matters (PM_10_ and PM_2.5_), nitrogen oxide (NO_x_), sulfur dioxide (SO_2_), carbon monoxide (CO), CO_2_, methane (CH_4_), non-methane volatile organic compound (NMVOC), and hydrocarbon (HC) under different operational modes, i.e., hoteling, maneuvering, and reduced speed zones. The results indicated that CO_2_ was the highest contributor with a proportion of 92%, NO_x_ 5%, and SO_2_ 1.5% for all three operational modes. Moreover, the results indicated that container ships account for 64% of overall emissions, followed by tankers for 24%. Regarding the monthly trend, the findings revealed that November and December had the highest emission rates, with over 20% of the total emissions recorded. This study’s findings will assist stakeholders and policymakers to prioritize maritime emissions in developing countries.

## 1. Introduction

Global warming and climate change arising from anthropogenic greenhouse gas (GHG) emissions are regarded as the greatest threats to the 21st century [1]. Transportation has the highest dependence on fossil fuels of any sector [2,3], and accounts for 37% of CO_2_ emissions [4]. The increase in port traffic over the past two decades has made shipping one of the most polluting sectors in the world [5]. Maritime transportation is responsible for around 940 million tons of CO_2_ and approximately 3% of global emissions annually [6]. The third International Maritime Organization (IMO) GHG research reveals that if mitigation measures are not implemented by 2050, CO_2_ emissions are expected to rise by 50% to 250%, which is quite alarming [7]. Maritime emissions can be easily transmitted over hundreds of kilometers inland, and their effects can be observed on both local and global scales, posing a considerable risk to human health. In reality, the emissions from ships may have a negative impact on air quality during hoteling, maneuvering, and transiting along the coast [5]. According to previous research, ship engine combustion emits 450 distinct types of air pollutants. Key ship-source air pollutants include GHGs, CO, NO_x_, SO_2_, and PM. In the coastal regions of Europe, East Asia, and South Asia, 60,000 cases of cardiac and lung cancer are tragically diagnosed each year due to PM emissions from shipping activity [8]. Air pollution is a global challenge that has serious consequences for human health, quality of life, port sustainability, and the country’s economy [9,10,11,12]. The global climate risk index ranks Pakistan as the seventh most vulnerable nation to climate hazards [13]. Air pollution has been attributed to 11 million premature deaths in Pakistan, out of 153 million premature deaths worldwide [14]. On 30 October 2019, the air quality index (AQI) in Lahore, Pakistan’s second-largest city, was 484—significantly higher than the “hazardous” threshold of 300. Pakistan’s 23.6 million automobiles account for 58% of all NO_x_ emissions [9,15,16]. The most recent database on air quality from the World Health Organization (WHO) demonstrates that 97% of affected cities with more than 100,000 residents are in low- and middle-income countries [15]. According to estimates by the World Bank, Pakistan’s annual disease burden from ambient air pollution causes around 22,000 adult premature deaths and 163,448 disability-adjusted life years (DALYs) [17]. Recently, a severe heatwave dominated Central and Upper Sindh, Pakistan due to climate change, and the temperature increased from 45 to 48 °C [18].

Maritime transportation is a key part of the international supply chain, and it enables regions and countries to prosper economically. It is an effective and inexpensive mode of transportation [19]. It is a major mode of carrying cargo and it contributes 90% of global trade by volume; hence, shipping emissions have a significant contribution to global emissions [8,20]. The United Nations Sustainable Development Goals (SDGs) 2030 agenda, which comprises seventeen SDGs, is an urgent call to action for all nations, developed and developing alike. Goal 13 is concerned with climate change. In response to this agenda, the IMO started supporting SDG 13 (climate action) to tackle climate change and developed a strategy to reduce GHG emissions from ships in accordance with the Marine Environment Protection Committee’s 72nd and 74th meetings. The IMO declared “World Maritime Theme 2022: New Technologies for Greener Shipping” in July 2021. The theme was connected to SDG 13, climate action: sustainable use of oceans, seas, and marine resources; SDG 9: industry, innovation, and infrastructure; and SDG 17: the crucial role of partnerships in achieving these goals. In 2018, during the 72nd meeting of the Marine Environment Protection Committee, the IMO adopted a resolution to reduce shipping emissions by at least 50% and CO_2_ emissions by at least 70% by 2050 [21].

In summary, a comprehensive literature analysis reveals that no study has been conducted to estimate shipping emissions in the context of Pakistan. It has been observed that a large number of studies have focused on road traffic emissions in Pakistan, but maritime transportation has been largely neglected. This mode of transportation has profound consequences on the port’s surrounding area and has substantially degraded the natural environment. Pakistan is also a signatory to the 2030 United Nations SDGs agenda. Therefore, in order to meet internationally agreed mitigation levels, the shipping industry must make substantial reforms to its emissions footprint. It is widely acknowledged that Pakistan has shown a strong interest in mitigating GHG emissions by implementing several significant initiatives, such as the Billion Tree Project and an ambitious plan to convert fossil fuel vehicles to electric and renewable sources [22,23]. In addition, to combat GHG emissions and cope with the above catastrophic and pressing concerns, it is urgently necessary to estimate shipping pollutants emissions and their relative influence, by conducting empirical studies. Thus, this study intends to bridge the above lacuna by estimating shipping emissions of various toxic pollutants at the port of Pakistan. To the best of the authors’ knowledge, this is the first study that considers shipping emissions of Pakistan port, namely MBQP.

The objective of this study was to estimate the various anthropogenic pollutants of ships (tankers, container ships, bulk carriers, and general cargo ships) emissions including PM_10_ and PM_2.5_, NO, SO_2_, CO, CO_2_, CH_4_, NMVOC, and HC under different operational modes, i.e., hoteling, maneuvering, and reduced speed zones at MBQP Pakistan. The inventory data were used from January 2020 to December 2020 to estimate emission patterns in the seventeen port terminals. This study employed an activity-based method to estimate the various anthropogenic pollutants. Moreover, this study calculated the emission social cost of each pollutant. The study not only sheds light on the existing emissions arising from ship activities in MBQP, Pakistan, but also highlights the inadequacies in port operations. Moreover, this study will assist policymakers and governments to formulate countermeasures for ship emissions.

## 2. Literature Review

There is a growing body of literature on shipping emissions and coastal pollution worldwide. Most of the studies are conducted in developed countries. In this context, Coello et al. [24] calculated air emissions from the UK fishing fleet using an automatic identification system (AIS)-based technique. The findings of the study showed estimates of 295.7 kilotons of fuel being utilized, and 914.4 kilotons of CO_2_ being emitted between May 2012 and May 2013. In addition, McArthur and Osland [25] investigated ship emissions and computed the external costs of pollution. Ship emissions are anticipated to cost between 10 and 21.5 million euros per year. Moreover, Papaefthimiou et al. [26] undertook a study to calculate exhaust pollutants for international cruise ships to and from 18 ports in Greece in 2013, for maneuvering and hoteling purposes. The results revealed that during hoteling around 89.2% of all emissions were released. Goldsworthy and Goldsworthy [27] conducted a study in Australia and their results indicated CO_2_ was the highest contributor (1,417,418 tons), followed by NO_x_ (21,468 tons), and SO_2_ (21,099 tons). Lonati et al. [28] evaluated the effect of atmospheric emissions from port operations on local air quality for a new port project in the Mediterranean Sea. According to the emissions evaluation, NO_x_ emissions were the most consistent emissions from ships.

On the other hand, from the context of developing countries, in Shanghai Yanshen Port, China, Song [29] investigated both in-port ship emissions and the social impact of these emissions. The findings showed that CO_2_ was the highest contributor (578,444 tons) followed by CH_4_ (10 tons). Similarly, Song and Shon [30] calculated potential emissions from ships passing through the largest port in South Korea, the Busan Port, over three years in 2014. The biggest pollution emitters were container ships, which accounted for 45–67% of overall emissions. Moreover, Fan et al. [31] developed a model to calculate shipping emissions within 400 km of the coast using AIS data from the Yangtze River Delta (YRD) and the East China Sea. Their results showed that within 100 and 200 km of the coast, the ship’s emissions were over 60 and 85%, respectively. Ships emitted SO_2_ and NO_x_ significantly higher than land-based sources. Ng et al. [32] established a new exhaust emission inventory of ocean-going vessels (OGVs) for Hong Kong using AIS data to calculate typical main engine load factors, by characterizing vessel speed and operation mode. In 2007, it was determined that container ships were the most polluting vessel type, contributing 9886 tons of SO_2_, 11,480 tons of NO_x_, 1173 tons of PM_10_, 521 tons of VOC, and 1166 tons of CO, or around 80–82% of the total emissions. Likewise, Wan et al. [33] calculated emissions at different ports in mainland China such as Pearl River Delta, Bohai Bay, and Yangtze River Delta. The results showed that container ships accounts for 50% of all emissions at cruising stage. In addition, Deniz et al. [20] calculated PM, HC, CO_2_, SO_2_, and NO_x_ emissions from 7520 ships. The findings indicated that the majority of emissions are generated during hoteling. Furthermore, Saraçoğlu et al. [34] estimated ship emissions of exhaust gases. Container ships were responsible for the greatest amount of pollution. In terms of operational modes, cruising and hoteling produced the most emissions. Yau et al. [35] calculated Hong Kong’s ocean-going vessels’ exhaust emissions. The study findings indicated that container ships accounted for more than 70% of the total NO_x_, SO_2_, and PM_10_ emissions. In terms of operational modes, 70% of total emissions were attributed to reduced speed zones and hoteling. Kuzu et al. [36] analyzed and estimated shipping emissions in Bandirma, Turkey, where ships are berthed. PM_10_, NO_x_, SO_2_, and CO emissions were found to be 182.4, 7996.6, 1681.6, and 239.6 tons, respectively. In the context of Pakistan, the vast majority of studies have been undertaken at the city level to estimate toxic pollutants emissions, especially those from road transportation and industries [37,38,39,40,41,42,43,44]. In addition, a recent study on MBQP coal power plant for estimating health risks of the fine particulate matter showed that there are serious health concerns due to emissions from the power plant. These emissions have a direct impact on port employees’ health and ultimately affect the environmental sustainability of MBQP [45].

In summary, the literature demonstrates a variety of approaches for estimating shipping emissions. The top-down approach [46,47], the bottom-up approach [5,20,28,30,31,32,34,35,48], and the hybrid approach (top-down and bottom-up approach) [47] are used to estimate shipping emissions in a different geographical context. On a global scale, the top-down method of estimating ship exhaust emission inventories can yield relatively accurate results; however, on a regional scale, it underestimates the amount of ship exhaust emissions [47,49]. The bottom-up method calculates ship emissions based on the activity trajectories of ships. To acquire accurate inventories of ship exhaust pollution, an activity-based model is utilized to determine the ship exhaust emission of a single ship. In terms of computing precision and temporal resolution, the bottom-up method is preferable to the top-down method when calculating regional ship emissions [47,49]. In addition, numerous studies have been undertaken in the context of developed countries, such as those in Europe, East Asia, West Asia, Australia, and New Zealand. However, there are few studies in the context of South Asia, and shipping emissions in Pakistan are largely ignored. Pakistani ports are essential to international maritime trade. Therefore, this study is intended to bridge the mentioned lacuna in the literature by estimating shipping emissions of toxic pollutants in a Pakistan context, since it will provide an overview of Pakistan’s maritime and significant guidance and direction.

## 3. Materials and Methods

### 3.1. Study Area

**Figure 1:**
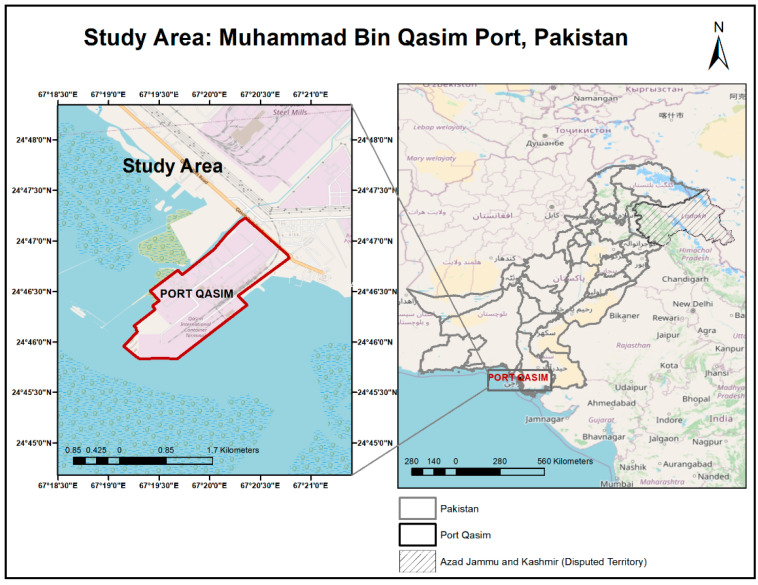
Study area, Muhammad Bin Qasim Port, Pakistan.

Pakistan, located in the center of the Indian Ocean, is a renowned coastal region that handles over 95% of its trade through its seaports, including Karachi, Qasim, and Gwadar [50]. Muhammad Bin Qasim Port (MBQP) is also known as Port Qasim. It is Pakistan’s second busiest and deepest seaport. It is located 28 miles southeast of Karachi, in the Indus delta region. It has a 49-km-long navigational channel. This port can accommodate ships with a depth of 13 m and a length of 347 m [50]. It consists of a total of 17 terminals, categorized into import terminals, export terminals, and import/export terminals. At the MBQP, cargo handling is performed by the private sector. Cargo Handling Companies (CHCs) at the marginal wharf are equipped with forklifts, cranes, hoppers, and evacuators for the handling of general and bulk cargoes. MBQP is well-connected to the transportation infrastructure of the country. A 14-km-long railway line and specialized train station connect the port to the national rail network [51]. According to the Ministry of Maritime Affairs Pakistan yearbook 2019–2020, MBQP achieved a throughput of over 51.017 million tons which is 4% more than in 2018–2019. During the year 2018–2019, the Pakistan Seaborne Trade stood at 92.818 million tons, out of which MBQP handled 51.017 million tons of cargo, which is represented by around 55% of MBQA’s share [52,53]. The volume of imported cargo during July–June 2019–2020 stood at 43.509 million tons, against the 41.878 million tons handled during the corresponding period 2018–2019, showing an increase of 3.89%. The exported cargo handled was 7.508 million tons during the twelve months of the financial year 2019–2020, as compared to 7.152 million tons handled during the corresponding period 2018–2019, showing an increase of 4.96% [52,53]. The study area of MBQP is depicted in Figure 1.

### 3.2. Data Collection

Comprehensive ship activity data were obtained from the MBQP database [50] which includes ship-related data such as tonnage, arrival time, departure time, and import/export information. The survey information and operational data, such as ship names, arrival, and departure information (used to compute hoteling times) and maneuvering times, as well as ship names/identification numbers, date of construction, gross weight, and gross tonnage (GT), were collected from shipping databases [54], as well as port authorities [50]. During the study period, a total of 1438 ships arrived at MBQP, including bulk carriers, tankers, container ships, and general cargo ships [50]. This study analyzed the 1438 ships for which data were available, and 271 were excluded due to incomplete data. The sample vessel profiles for the years January 2020 to December 2020 are attached in Appendix A (Table A1). 

### 3.3. Research Methodology

#### 3.3.1. Emission Inventory Methodology

Top-down and bottom-up are the two most common methods for creating ship emission inventories. The top-down method, also known as the fuel-based technique, is used for calculating ship exhaust emission inventories by employing total fuel usage and fuel emission factors [46,47]. On the other hand, the bottom-up method, also known as the activity-based method, is utilized to collect data on individual ship activities, and the sum of each ship’s energy consumption and emissions yields the total emissions [5,33,49]. In this study, the bottom-up method was employed to estimate different emissions as a result of shipping activities during the year 2020. The Tier 3 approach from the 2016 European Monitoring and Evaluation Program (EMEP) and European Environment Agency (EEA) air pollutant emission inventory manual was used to estimate air pollutant emissions from ships in this study. The bottom-up method relies on ship movement data and applies emission factors to a specific ship activity. The emission factors are used to establish a relationship between the amount of a particular pollutant emitted and the amount of energy consumed by the ship’s engines during a specific activity (considering the engine’s operating parameters). The study considered the following activities: (i) hoteling, considering all the time the ship was inside the port. In the context of shipping emissions studies, hoteling represents a stage where ships spend time at berths in order to fulfill port operations (loading–unloading of cargo). In the hoteling phase, the ship’s main engines are turned off, and auxiliary engines work to generate power to ensure the ship’s onboard services. The second activity is (ii) maneuvering, which considers the average time when the ship is inside the port during maneuver operations (provided by port authorities). The third activity is the (iii) reduced size zone (RSZ) mode, in which the vessel approaches the coast while reducing its speed. For every ship call, the number of air pollutants (PM_10_, PM_2.5_, NO_x_, SO_2_, CO, CO_2_, CH_4_, NMVOC, and HC) produced during hoteling, maneuvering, and RSZ mode was evaluated. The method for calculating emissions is based on the installed power and time spent throughout the various phases of navigation. Port emissions are computed using the following equations for each vessel and pollutant (1)–(4).

(1) $$E_{i,j,f}\;=\;\sum_{p}[T_{p}\sum_{e}(P_{e}*LF_{e}EF_{e,i,j,f,p})]$$

where;

— *E* = emissions;
— *EF* = emission factor (g/kWh);
— *LF* = engine load factor (%);
— *P* = engine power (kW);
— *T* = time spent (h);
— *e* = engine category (main, auxiliary);
— *i* = pollutant;
— *j* = engine type (slow-, medium-, high-speed diesel);
— *f* = fuel type (BFO, MDO); and
— *p* = operation mode of trip (port, maneuvering, anchorage).

(a) Emissions at hoteling stage

In the first stage of estimation, emissions at the hoteling stage are calculated using Equation (2).

(2) $${\mathrm{Emissions}}_{\mathrm{hott}}\;=T_{\mathrm{hott}\;}\left(ME*LF_{ME}*EF_{ME}+AE*LF_{AE}*EF_{AE}+B*EF_{B}\right)$$

where;

— *T*_hott_ = the time spend by vessels at the berthing stage (h);
— *ME* = the maximum main engine power (kW);
— *LF_ME_* = the load factor of the main engine (%);
— *AE* = the auxiliary engine power (kW);
— *LF_AE_* = the load factor of auxiliary engine (%);
— *B* is the auxiliary boiler energy default; and
— *EF* = emission factors associated with each engine type in hoteling mode (g/kWh);

(b) Emissions at maneuvering stage

The emissions at the maneuvering stage are calculated using Equation (3).

(3) $${\mathrm{Emissions}}_{man}\;=\;T_{man}\left(ME*LF_{ME}*EF_{ME}+AE*LF_{AE}*EF_{AE}+B*EF_{B}\right)$$

where;

— *T_man_* = the time spend by vessels at maneuvering stage (h);
— *ME* = the maximum main engine power (kW);
— *LF_ME_* = the load factor of the main engine (%);
— *AE* = the auxiliary engine power (kW);
— *LF_AE_* = the load factor of auxiliary engine (%);
— *B* is the auxiliary boiler energy default; and
— *EF_ME_*, *EF_AE_*, and *EF_B_* are emission factors associated with each engine type in maneuvering mode(g/kWh).

(c) Emissions at reduced speed zone

Reduced speed zone emissions from different ship categories are calculated using the average time spend in the operational mode. The mathematical form of the reduced speed zone is shown in Equation (4).

(4) $${\mathrm{Emissions}}_{RSZ}\;=\;T_{RSZ}\left(ME*LF_{ME}*EF_{ME}+AE*LF_{AE}*EF_{AE}\right)$$

where;

— *T_RSZ_* = the time spend by vessels at the reduced speed zone stage (h);
— *ME* = the maximum main engine power (kW);
— *LF_ME_* = the load factor of the main engine (%);
— *AE* = the auxiliary engine power (kW);
— *LF_AE_* = the load factor of auxiliary engine; and
— *EF_AE_* are emission factors associated with each engine type in the RSZ mode.

(d) Emission social cost

Emission social cost is defined as the sum of environmental and social costs caused by the shipping activities and moments in the port region. This cost comprises biodiversity loss, crop loss, health impacts, and material damages [28,35]. The social cost of shipping emissions is calculated as the sum product of the emission amount (ton) and the emission’s social cost factor ($/ton).

(5) $$\mathrm{Social}\;Cost=\sum Emission_{i}*SCF_{i}$$

where;

— *Social Cost* = total calculated monetary value in dollar ($);
— *Emission* = emission totals per pollutant type (a);
— *SCF* = value of pollutant ($/ton); and
— *i* = pollutant type.

Due to the lack of studies on shipping emissions in the context of Pakistan, for the current study, we borrowed the emission social cost factors (SCF) from a previous study [28,35].

#### 3.3.2. Research Framework

**Figure 2:**
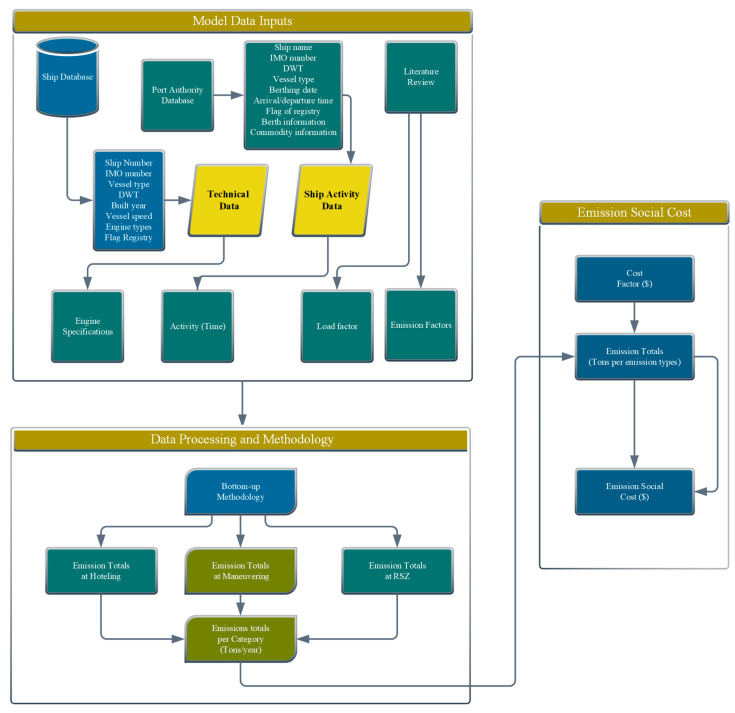
Schematic diagram of the study.

In this research, the initial step was the data input, and the technical data for each ship such as ship type and main engine–auxiliary engine characteristics were obtained from ship information databases and manufacturer’s manuals. Moreover, ship-related data such as tonnage, arrival time, departure time, and import/export information were obtained from the port authority’s databases. After a thorough review of the literature, information on activity-related factors coefficients such as the load factors and emission factors were obtained from reliable comprehensive emission inventory studies. The second step involved data processing using standard procedures to estimate emissions per category under different operational modes using the bottom-up method. The third phase involved the assessment of the social cost based on emission totals by category and social cost factors associated with each emission type, as depicted in Figure 2.

#### 3.3.3. Engine Powers and Load Factors

Since acquiring ship engine-related data itself is a tough task to achieve, for this study there were some missing data related to a few vessels. The maximum engine powers of the ME, AE, and auxiliary boilers, as well as the load factors for the engines in different operating modes, were obtained from default tables [5]. ME maximum powers are a function of GT (gross tonnage) in accordance with the EMEP/EEA Guidebook 2016 which is attached in Appendix A (Table A3) [55]. Table 1 indicates the expressions for the calculations of the vessel’s main engine using gross tonnage and auxiliary engine power ratios by different types of ships. Emission factors (EF) are heavily reliant on engine/fuel type profiles and the sulfur content of the fuel. Emission factors for a case study of MBQP were taken from other previous studies and are attached in Appendix A (Table A3) [55,56,57,58,59]. In this study, engine/fuel type profiles were extracted from the Entec UK Limited LMIS database in 2010 [59]. The AE-rated powers for missing data were obtained from ME-to-AE ratios by ship type, as developed by Trozzi [60].

## 4. Results and Discussion

This study estimated the emissions of CO_2_, PM_10_, PM_2.5_, NO_x_, SO_2_, CO, CH_4_, NMVOC, and HC during the reduced speed zone, maneuvering, and hoteling for all ships arriving at MBQP from January to December 2020, as shown in Table 2. A total of 1709 ships were anchored at MBQP during this period, out of which 1438 ships were studied for emission inventory and 271 were excluded due to incomplete data. In this study, there were four ship categories including 481 container ships, 321 bulk carriers, 579 tankers, and 53 general cargo ships. These ships were anchored at different terminals in MBQP (a total of 17 terminals) [50]. It can be seen in Table 3, that during the year 2020, the maximum number of port calls were from tankers, as Pakistan imports, a bulk quantity of oil and gas products monthly, followed by bulk carriers.

**Figure 3:**
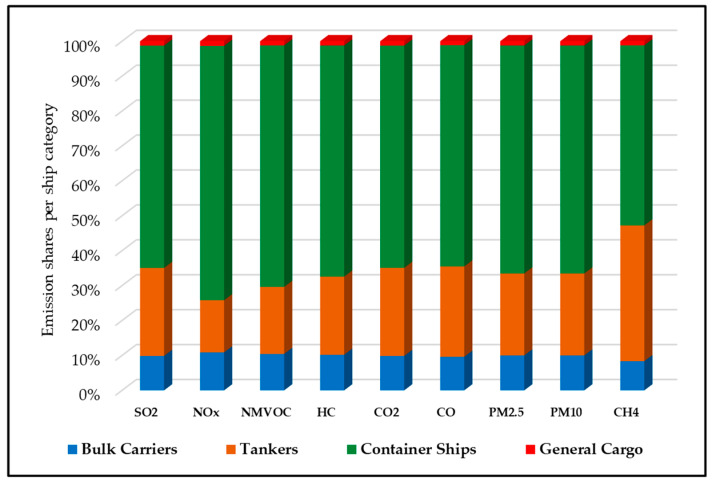
Ship emissions of various ship types at MBQP in 2020.

Figure 3 indicates different types of ship emissions. It can be seen that container ships are the largest pollutant emitters, emitting a total of 76,372 tons of pollutants per year, followed by tankers which are emitting approximately 29,422 tons per year. One of the reasons container ships account for maximum emissions is the engine powers and emission factors associated with residual oil (RO) fuel. This result is consistent with a previous study conducted in China’s ports, which indicated that 50% of all emissions were from containers [33]. Another similar study conducted in Turkey revealed that containers contribute to 92% of all emissions [61]. Likewise, a study conducted in South Korea revealed that cargo ships were responsible for between 45 and 67% of total emissions [30].

The emissions from different engine types are significantly heterogeneous. Previous studies also showed that the main source of emissions is the ship’s main engine due to load and high power [33]. As in this study, the cruising stage was not considered, so the auxiliary engine emissions accounted for approximately 70% of total emissions, followed by boiler emissions which accounted for 28% of total emissions. The emissions totals for each engine type can be seen in Table 3. The study results revealed that CO_2_ had the highest share in emission totals (more than 92% of the total), while NO_x_ emissions (5% of the total) were the second highest, followed by SO_2_ emissions (1.5–1.8%) and PM_10_ and PM_2.5_ emissions (0.3% of total), and CH_4_ accounted for the lowest share in the total estimated emissions. This result is in line with a previous study which showed that CO_2_ emissions were the highest share as compared to other pollutants [36,61]. The results of a previous study Indicated that the emissions from the main engine were three to four times more than those from the auxiliary engine and boiler when cruising, hoteling, and maneuvering [33]. We did not include the cruising operational mode in our case study due to a lack of data. This is because the main engine had fewer emissions than the auxiliary engine. This finding is aligned with a previous study conducted at South Asian ports [62].

**Figure 4:**
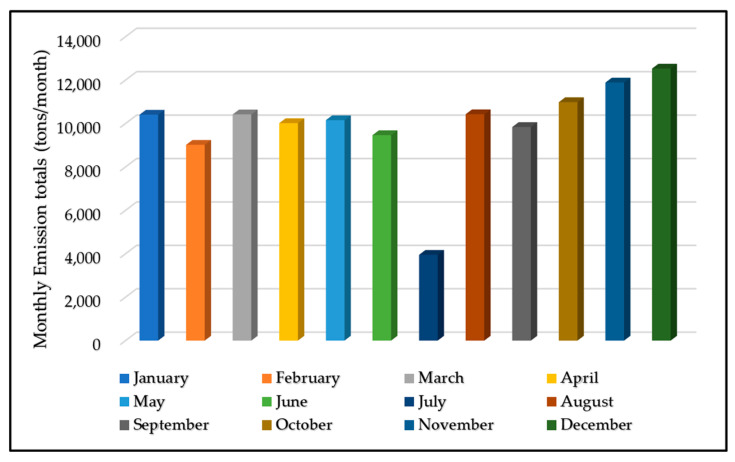
Monthly emissions patterns.

The MBQP authority’s statistics indicate that the monthly ship arrivals were varied. Figure 4 shows the monthly emissions patterns at MBQP from January 2020 to December 2020. The months of November and December recorded a maximum number of ship calls totaling 10.5 and 9.9% of total emissions, respectively, while July had the least number of calls, with 3% of total emissions. The period from September to December 2020 accounted for 37–38% of total emissions during the year. This is attributed to the number of ship calls during these four months being relatively high as compared to other months. The results of the current study are in line with the previous study conducted in China. The findings of their study indicated that most of the emissions were recorded between September and November. During the Chinese spring festival period, the emissions were reduced significantly. It was observed that during November, estimated container ship emissions were 1,385,815 tons, which is 2.28 times that of January’s 607,772.9 tons [33].

### 4.1. Emissions at Three Different Operational Stages

During the hoteling, operational mode emissions of NO_x_, SO_2_, PM_10_, PM_2.5_, CO, NMVCO, CH_4_, CO_2_, and HC were estimated for different ship types that arrived at MBQP in the year 2020. Regarding emissions by type, those of CO_2_ were found to be dominant. In this study, a total of 106,970.9 tons/year of CO_2_ was emitted during hoteling, followed by NO_x_ emissions (6027.7 tons/year), SO_2_ emissions (1819.9 tons/year), NMVOC (183.9 tons/year), CO (112.1 tons/year), and PM_2.5_ and PM_10_ emissions (177.1 and 193.2 tons/year) during the study period, as depicted in Table 4. Contrarily, the emission of CH_4_ was recorded as less than 0.1% of total estimated emissions. Generally, in the hoteling stage, the main engine is turned off, while the auxiliary engine and boiler are being operated [62]. Compared with the previous study, similar patterns can be seen in terms of emission quantity. The emissions quantity was higher during hoteling compared with the reduced speed zone and maneuvering stages [33]. Previous studies also indicated the fact that container ships and bulk carriers are the largest emitters of pollutants. These two ship categories have high-rated power engines; hence, emissions are high. A study conducted in Piraeus Port (Greece) also showed that hoteling emissions (89.2%) are larger than maneuvering emissions (10.8%) for all ship categories. NO_x_ emissions are the largest contributor, whereas SO_2_, NMVCO, and PM emissions follow with considerably lower emissions [26].

A previous study of Alsancak Port considered four operation modes, and their results showed that the hoteling stage had a higher emission than the maneuvering stage. It is necessary to build infrastructure and control the emissions within the port region [61]. This is attributed to the fact that the emissions would also be increased significantly, as import totals increased significantly. As a result, developing ways to minimize ship traffic density in the inner bay and shorten the hoteling duration is advocated as part of emission reduction initiatives. In addition to the above suggestions, replacing the ship’s energy source in ports (cold ironing) is recommended to reduce the number of pollutants released into the atmosphere [61].

During the maneuvering stage, CO_2_ was the highest contributor by mass, with a total value of 1735.9 tons/year as shown in Table 4. Among the other pollutants, NO_x_ has the second-highest mass share (111.4 tons/year). Previous studies showed that hoteling stage activities accounted for 90% of NO_x_ emissions while the remaining share is due to the maneuvering stage [26]. The third highest contributor to shipping emissions was SO_2_ during the maneuvering stage (29.5 tons/year). PM_10_ and PM_2.5_ emissions (3.3 and 3.1 tons/year, respectively) and emissions of CH_4_ were found the lowest emitter during the maneuvering stage at MBQP as shown in Table 4. In general, the highest emissions were estimated in the hoteling stage, followed by the maneuvering stage, where ships spend less time as compared to hoteling time. A previous study undertaken for three ports in Portugal followed an analogous trend for emission totals during the maneuvering stage, i.e., CO_2_ was the highest followed by NO_x_ [5].

During the reduced speed zone operation mode, CO_2_ was the highest contributor with a total value of 1520.1 tons/year, followed by NO_x_ emissions (99.1 tons/year) and SO_2_ (25.8 tons/year) as depicted in Table 4. A previous study conducted in Nordic Port, Norway and their findings showed that around 50% of emissions from oceangoing vessels occur at berth, while other operation modes such as RSZ operation stage and maneuvering account for lesser emissions totals. Moreover, they suggested that the implementation of onshore power, and encouraged the use of liquid natural gas (LNG) in order to control emissions. Their results suggested that onshore power provides reductions of up to 15% in NO_x_ and CO_2_ emissions [63].

### 4.2. Ship Emissions Studies Comparison with Other Ports

The emissions from four ship categories (container ships, bulk carriers, tankers, cargo ships) of this study were compared to those emission inventory studies for other ports in different countries. All the studies utilized for comparison used the activity-based method to estimate ship emissions, despite the calculations having different geographic locations, ship types, port activities/operations, and pollutants that were taken into account. Regardless of these limitations, some comparisons can be beneficial. Table 5 depicts a comparison with previous emissions’ inventory studies.

Ship emissions totals for this study were far lower than those estimated by Wan et al. [33], as three different regions in Mainland China have some of the world’s busiest ports. They estimated emissions for a total of 161,080 ships (4 operational modes) for three different regions, while the current study estimated emissions for a single port (3 operational modes). Considering the ship category contributing maximum emissions, both studies showed that container ships are the largest pollutant emitters. In comparison with Alsancak Port, Turkey [61], it was found that NO_x_ and SO_2_ totals were lower than the current study, but both studies showed that the hoteling operation mode had the highest estimated emission. Kuzu et al. [36] estimated that concentrations for NO_x_ were the highest (7997 tons/year) as compared to CO_2_ (272,301 tons/year), while the current study estimated 6232.5 tons/year of NO_x_ and 110,227.0 tons/year of CO_2_ for the study period. This is due to the fact that the above study considered 1577 ships and our study considered 1438 ships. In the study by Buber et al. [7], emission totals for Izmir Bay region were 64,222.2 tons per year, while our study estimated a total of 119,107.9 tons/year. The emissions trend was found to be largely analogous, as CO_2_ was the highest contributor followed by NO_x_ and SO_2_. In addition, the current study is an initial step to draw the attention of researchers and petitioners toward the importance of port environmental sustainability and port emission control policies in emerging economies, such as Pakistan.

### 4.3. Emission Social Cost

The emission social cost is defined as the total of the social and environmental expenses as a result of shipping activities in the port area [29]. This emission social cost consists of crop loss, health impacts, and material damage of biodiversity loss [36]. Due to the unavailability of studies on marine emissions in the context of Pakistan, for this case study, we utilized the social cost factors (SCF) from previous studies [29,36]. The estimated social costs for the current study are given in Table 6. These values were obtained by using the earlier Equation (5).

The current study results showed that NO_x_ contributed to the maximum emissions after CO_2_. The social cost of NO_x_ per ton ranged between 269~58,300 USD/ton, which is attached in Appendix A (Table A2). The social cost of NO_x_ was estimated at $66.63 million; thus, it ultimately rendered NO_x_ the most dominant and costliest in terms of the burden on society and the environment. After NO_x_, the social cost of CO_2_ was estimated at $3.19 million. The reason is that the SCF for CO_2_ was much lower than NO_x_. NO_x_ took 54.1% of the total social cost because its social cost factor is higher as estimated by previous studies [64,65]. In the past, several studies have been conducted to estimate the social costs of shipping emissions. Tovar and Tichavska [66] investigated environmental costs due to shipping activities for three different regions including Saint Petersburg, Las Palmas, and Hong Kong. This concludes that Hong Kong had the greatest social cost values due to its immense ship traffic. Kuzu et al. [36] studied the environmental cost of shipping emissions for Bandirma Port in Turkey. The total estimated social cost was approximately €41.1 million. Dragović et al. [67] also investigated the social costs of shipping activities (cruise ships) in two ports in Croatia. This study was conducted on a total of 436 cruise ships. The results showed that the total social costs for Kotor Port were €10.8 and that of Dubrovnik Port €23.7 million, which is aligned with the current study. McArthur and Osland [25] studied the atmospheric emissions and their environmental cost of the Port of Bergen, Norway. The total estimated environmental costs were €10.58 million, approximately. Song [29] conducted a study on the shipping activities of Yangshan Port, Shanghai, China, and calculated the social cost and eco-efficiency. During the study, 6518 container ships were investigated, and the estimated total social cost was $287 million, approximately. It is intuitive that due to greater maritime traffic in Yangshan Port, China, the social cost will be higher as compared to MBQP of Pakistan. In comparison, NO_x_ is the costliest pollutant, followed by SO_2_ and particulate matter (PM_2.5_, PM_10_).

## 5. Conclusions

Maritime transportation can play a crucial role in achieving sustainable growth due to its significance to the world economy. This study aimed to estimate the various anthropogenic pollutants of ships’ (tankers, container ships, bulk carriers, and general cargo ships) emissions including PM_10_ and PM_2.5_, NO_x_, SO_2_, CO, CO_2_, CH_4_, NMVOC, and HC under different operational modes, i.e., hoteling, maneuvering, and reduced speed zone at MBQP Pakistan. This study used the inventory data from January to December 2020 to estimate emission patterns in the seventeen port terminals. This study employed an activity-based method to estimate the various anthropogenic pollutants. Moreover, this study calculated the emission social cost of each pollutant, which is the sum product of pollutant emissions and emission’s social cost factors.

The contribution of this study was to analyze the shipping of toxic pollutants in the context of a developing country such as MBQP, Pakistan. The findings of this study indicated that container ships account for 64% of total emissions, followed by tankers for 24%. The results indicated that CO_2_ was the highest contributor with a proportion of 92%, NO_x_ 5%, and SO_2_ 1.5% for all three operational modes. Regarding the monthly trend, the findings revealed that November and December had the highest emission rates, with over 20% of the total emissions recorded. In terms of social cost, NO_x_ contributed to the maximum emissions. The social cost of NO_x_ was estimated at $66.63 million, thus making NO_x_ the most dominant and costliest in terms of the burden on society and the environment. After NO_x_, the social cost of CO_2_ was estimated at $3.19 million. In addition, the current study revealed that the emission quantity was highest during hoteling compared with the reduced speed zone and maneuvering stages. This is attributed to the fact that the emissions increase significantly as import totals increase significantly. As a result, developing ways to minimize ship traffic density in the inner bay and shorten the hoteling duration is advocated as part of emission reduction initiatives. In addition to the above suggestions, replacing the ship’s energy source in ports (cold ironing) is recommended to reduce the number of pollutants released into the atmosphere.

In emerging economies, there is a lack of the latest technology interventions such as electric cargo handling equipment, electric quay cranes, and rubber tire gantry cranes (RTGs). It is therefore suggested that the latest technologies such as industry 4.0 technology could be provided in cargo handling at ports in order to mitigate emissions and be more beneficial to speed up transactions and complete port operations faster. In addition, the use of low-sulfur fuel or various alternative fuels may potentially effective way of reducing port emissions. Port authorities must encourage shipping companies to switch to environmentally friendly fuels by providing them with different incentives, such as initiatives implemented by Singapore, namely the Green Award, and the Netherlands, namely the clean shipping index, as well as incentives to shipping fleets such as a discount on port dues. Thus, port authorities must create new laws regarding ship fuels, such as requiring all hoteling vessels to have a sulfur concentration of 0.5% by mass and imposing carbon emission taxes. Emission Control Area (ECA) laws may have a significant impact on decreasing emissions in ports; therefore, port authorities must develop new regulations in conformity with international standards. This study conducted a meta-analysis by reviewing the SCF values in the existing studies and then used them in the context of Pakistan. Unfortunately, the local data shortage in Pakistan makes it difficult to conduct extensive research on the local SCFs and gain values in a range with a high confidence level. Therefore, forthcoming studies should consider air quality dispersion models and the human health impact of shipping pollution. This research is essentially a baseline for future marine studies, particularly the environmental aspects of ship activity at developing countries’ key ports such as Pakistan.

## Figures and Tables

**Table 1:** Regression equation of main engine power and auxiliary engine ratio.

Ship Type	Non-Linear Regression of 2010 World Fleet	AE Power Ratio
Bulk Carrier	14.755 × GT^0.6082^	0.30
Container Ship	2.9165 × GT^0.8719^	0.25
General Cargo	5.56482 × GT^0.7425^	0.23
Tankers	35.912 × GT^0.5276^	0.30

**Table 2:** Number of ships by types arriving at MBQP in 2020.

Types of Ships	Total Number
Bulk Carriers	321
Tankers	579
Container ships	481
General Cargo ships	53

**Table 3:** Ship emissions of different engine category (tons/year).

Engine Type	SO_2_	NO_x_	NMVOC	HC	CO_2_	CO	PM_2.5_	PM_10_	CH_4_
Main Engine	10.4	14.7	0.9	0.8	614.7	0.6	1.3	1.4	0.1
Auxiliary Engine	1304.4	6170.2	180.3	42.4	76,568.2	103.0	140.0	152.7	0.5
Boiler	560.5	49.6	9.3	9.3	33,044.1	12.4	41.8	45.5	0.6
Emissions Total	1875.3	6234.5	190.5	52.5	110,227	116	183.1	199.6	1.2

**Table 4:** Ship emissions of different operational modes (Tons/year).

Operation Mode	CO_2_	NO_x_	NMVOC	HC	SO_2_	CO	PM_2.5_	PM_10_	CH_4_
Hoteling	106,970.9	6027.7	183.9	50.4	1819.9	112.1	177.1	193.2	1.04
Maneuvering	1735.9	111.4	3.4	1.1	29.5	2.0	3.1	3.3	0.2
Reduced Speed Zone	1520.1	99.2	0.9	0.08	25.8	1.8	2.7	3.0	0.005

**Table 5:** Comparison with previous emission inventories studies.

Port	Inventory Period	Operation Analyzed	Pollutants Studies	Study	Emission (Tons/Year)
Muhammad Bin Qasim Port Pakistan	2020	RSZ, M, H	PM_10_, PM_2.5_, NO_x_, SO_2_, CO, CO_2_, CH_4_, NMVOC, and HC	Current Study	119,079
Bohai Bay, Yangtze River Delta, and Pearl River Delta China	2018	C, RSZ, M, H	PM_10_, PM_2.5_, NO_x_, SO_x_, CO, CO_2_, N_2_O, and HC	Wan et al. [33]	7,715,172.03 11,049,016.09 4,329,337.25
Izmir Bay, Turkey	2018	C, M, H	SO_2_, NO_x_, CO_2_, PM_10_, HC	Toz et al. [61]	20,425.8
Bandirma Port, Turkey	2018	H	PM_10_, NO_x_, SO_2_, and CO	Kuzu et al. [36]	282,685.3
Izmir Bay, Turkey	2018	C, M, H	SO_2_, NO_x_, CO_2_, PM, HC	Buber et al. [7]	64,222.6

Note: C: Cruising RSZ: Reduced speed zone M: Maneuvering: H: Hoteling.

**Table 6:** Ship emission social costs.

Pollutant Type	Emission Social Costs ($)
NO_x_	66,628,101.50
SO_2_	23,120,574
HC	156,712.50
CO_2_	3,196,583
CO	132,936
PM_2.5_	15,704,670
PM_10_	15,342,653
Total	124,282,230

## Data Availability

The data are available upon reasonable request from the first author of this paper.

## Appendix A

**Table A1:** Sample vessel profiles.

Ship Type	IMO No.	MMSI	Manufacturing Data	ME (kW)	AE (kW)	DWT	GT
Bulk Carrier	97,321 **	5,656,550 **	2014	8100	1552	57,945	32,750
Bulk Carrier	93,952 **	5,380,069 **	2009	8425	2527	53,428	31,094
Bulk Carrier	97,232 **	3,719,650 **	2019	9150	2745	63,539	36,353
Bulk Carrier	93,002 **	5,642,200 **	2005	8200	1552	55,862	30,822
Bulk Carrier	97,089 **	5,489,120 **	2015	8200	1560	57,811	32,399
Bulk Carrier	92,384 **	5,647,240 **	2002	7800	1340	52,383	30,303
Bulk Carrier	98,527 **	6,360,186 **	2019	8686	2605	63,555	35,832

Note: ME: Main engine; AE: Auxiliary engine; DWT: Deadweight tonnage; GT: Gross tonnage. **: Number continuity.

**Table A2:** Social cost factors (SCF).

Emission	Range (US$/ton)	Value Used in This Study (US$/ton)
CO_2_	15–42	29
CH_4_	250–2500	812
CO	160–3200	1146
PM_10_	2000–498,791	76,867
PM_2.5_	1000–554,229	85,771
NO_x_	269–58,300	10,687
SO_x_, SO_2_	379–64,997	12,329
HC	750–3824	2985

**Table A3:** Emission factors (g/kWh) for different engine types/fuel.

Engine	Phase	Engine Type	Fuel Type	Sulphur %	SO_2_	NO_x_	NMVOC	HC	CO_2_	CO	PM_2.5_	PM_10_	CH_4_
Main	RSZ	SSD	RO	2.70%	10.5	16.9	0.6	0.6	620	0.5	1.31	1.42	0.006
		SSD	MDO	1.00%	3.7	15.8	0.6	0.6	588	0.5	0.45	0.42	0.006
		SSD	MGO	0.50%	0.9	15.8	0.6	0.6	588	0.5	0.31	0.28	0.006
		MSD	RO	2.70%	11.5	13.0	0.5	0.5	677	1.1	1.43	1.32	0.004
		MSD	MDO	1.00%	4.1	12.3	0.5	0.5	645	1.1	0.47	0.43	0.004
		MSD	MGO	0.50%	1.0	12.3	0.5	0.5	645	1.1	0.31	0.29	0.004
		HSD	RO	2.70%	11.5	11.8	0.2	0.2	677	1.1	1.47	1.35	0.004
		HSD	MDO	1.00%	4.1	11.2	0.2	0.2	645	1.1	0.58	0.53	0.004
		HSD	MGO	0.50%	1.0	11.2	0.2	0.2	645	1.1	0.35	0.32	0.004
	Maneuvering Hoteling	SSD	RO	2.70%	11.6	4.7	2.5	1.8	682	1.0	1.32	1.43	0.012
		SSD	MDO	1.00%	4.1	4.7	2.6	1.8	647	1.0	0.44	0.47	0.012
		SSD	MGO	0.50%	1.0	4.7	2.6	1.8	647	1.0	0.29	0.31	0.012
		MSD	RO	2.70%	12.7	44.6	6.3	1.5	745	2.2	1.32	1.44	0.008
		MSD	MDO	1.00%	4.5	44.3	6.6	1.5	710	2.2	0.46	0.50	0.008
		MSD	MGO	0.50%	1.1	44.3	6.6	1.5	710	2.2	0.30	0.32	0.008
		HSD	RO	2.70%	12.7	40.6	8.2	0.6	745	2.2	1.32	1.44	0.008
		HSD	MDO	1.00%	4.5	40.1	8.6	0.6	710	2.2	0.46	0.50	0.008
		HSD	MGO	0.50%	1.1	40.1	8.6	0.6	710	2.2	0.30	0.32	0.008
Auxiliary	Maneuvering Hoteling	MSD	RO	2.70%	12.3	60.4	1.7	0.4	722	0.9	1.32	1.44	0.004
		MSD	MDO	1.00%	4.3	59.7	1.8	0.4	690	0.9	0.45	0.49	0.004
		MSD	MGO	0.50%	1.1	59.7	1.8	0.4	690	0.9	0.29	0.32	0.004
		HSD	RO	2.70%	12.3	47.6	1.7	0.4	722	1.3	1.32	1.44	0.01
		HSD	MDO	1.00%	4.3	46.8	1.8	0.4	690	0.8	0.45	0.49	0.01
		HSD	MGO	0.50%	1.1	46.8	1.8	0.4	690	0.8	0.29	0.32	0.01
Boilers	Maneuvering Hoteling	-	RO	2.70%	18.1	1.6	0.3	0.3	1067	0.4	1.35	1.47	0.02
